# Supplementary figures and images for: Perinatal exposure to a human relevant mixture of persistent organic pollutants: Effects on mammary gland development, ovarian folliculogenesis and liver in CD-1 mice
Source: PLoS One. 2021 Jun 10;16(6):e0252954. doi: 10.1371/journal.pone.0252954 (PMC8191980; doi:10.1371/journal.pone.0252954)

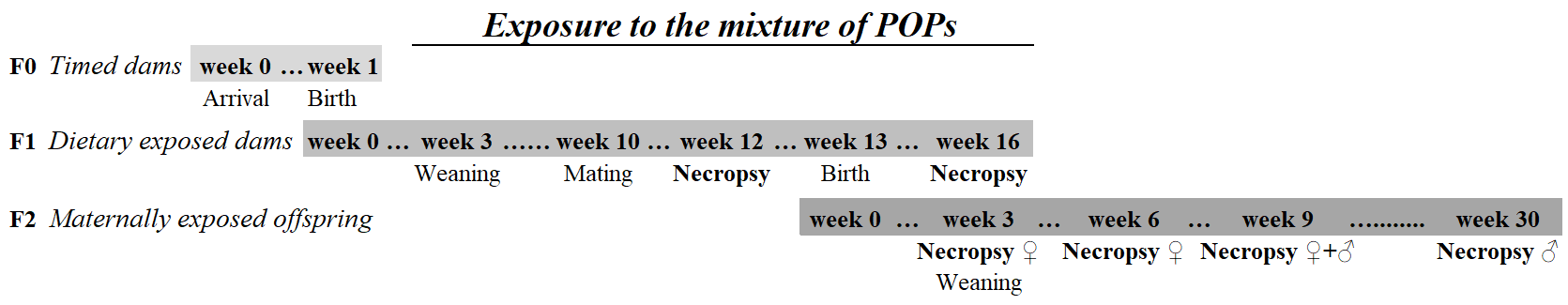

Supplement: S1 Fig — Timed-pregnant CD-1 dams (F0) gave birth to F1 dams at approximately 1 week after arrival. F1 dams were exposed to the mixture of POPs through the diet at Control, Low or High doses (0x, 5000x or 100 000x human estimated daily intake, respectively) from 3 weeks of age until termination during pregnancy (gestation d 17) or post-pregnancy (d 21 post-partum). F2 offspring were only exposed to the mixture in utero, through lactation and by nibbling on their mother’s food prior to weaning, and were terminated at 3 (females), 6 (females), 9 (females and males) or 30 (males) weeks of age. (TIF) [file pone.0252954.s001.tif]
