# Supplementary material for: Perinatal exposure to a human relevant mixture of persistent organic pollutants: Effects on mammary gland development, ovarian folliculogenesis and liver in CD-1 mice
Source: PLoS One. 2021 Jun 10;16(6):e0252954. doi: 10.1371/journal.pone.0252954 (PMC8191980; doi:10.1371/journal.pone.0252954)
Supplement: S1 File — (DOCX) [file pone.0252954.s002.docx]

# **S1 File. Supporting information on materials and methods**

## **Feed design**

Only HCB was detected in the control and ‘reference’ feed at concentrations of 2.24 and 8.04 ng/g, respectively. A deviation was detected between the nominal (estimated) and measured POP concentration in feed of ± 30% for most of the compounds. However, PCB-28 and the PFASs deviated with approximately 60-70% from nominal concentrations (see more information in [1]). The High dose aimed to be 20x higher than the Low dose, however, the ratio between the High and Low varied from 12 (for PCB-52) to 30 (for oxychlordane) times.

The Stockholm Convention on Persistent Organic Pollutants includes all PCBs, OCPs and BFRs in the mixture, in addition to PFOS and PFOA [2]. PFHxS is currently under consideration for inclusion, but the other PFASs (PFNA, PFDA and PFUnDA) have not yet been incorporated into the treaty, Nevertheless, all PFASs are known to be persistent in the environment and have the potential to bioaccumulate in animals and humans [3, 4].

## **Chemical analysis**

### **Chemicals**

Chemicals used for quantification included methanol, cyclohexane and acetone of HPLC quality (VWR International S.A.S, Radnor, Pennsylvania) and purified water was obtained from a Milli-Q Gradient A10 water system (Millipore, Bedford, MA, USA). Certified Reference Materials (CRM 2525, 350) were supplied by Cerilliant Corporation, Round Rock, TX, USA. Interlaboratory tests (human serum) were provided by Arctic Monitoring and Assessment Program (AMAP), Québec, Canada. All chemicals are routinely quality tested according to the accreditation requirements.

### **Analysis of PCBs, OCPs and BFRs**

Modifications to the methods by Brevik [5] and Polder and colleagues [6] are described below. Samples were protected from daylight during quantification to avoid degradation of the BFRs. <2.5 g pooled liver tissue was homogenized (using a T25 Ika Ultra-Turrax^®^) and spiked with internal standards (PCB-29, -112 and -207 obtained from Ultra Scientific, North Kingstown, USA, and BDE-77, -119, -181 and ^13^C_12_BDE-209 from Cambridge Isotope Laboratories Inc., Tewksbury, USA). Lipids were extracted twice with cyclohexane and acetone (3:2 ratio, obtained from VWR International S.A.S, Radnor, Pennsylvania) by ultrasonic homogenization, centrifugation and separation. Lipid content was determined gravimetrically in 1 mL aliquots, and lipid removal was performed by using ≥ 97.5% H_2_SO_4_ (Sigma Aldrich, Missouri, USA). Extracts were concentrated with a factor of approximately 10 by careful evaporation under N_2_, before gas chromatography (GC) analyses.

The quantification of PCBs (PCB-52, -101, -118, -138, -153, and -180) and OCPs (*p,p’*DDE, HCB, α-chlordane, oxychlordane and *trans*-nonachlor, and α-, β- and γ-HCH) was performed on a high-resolution GC (HRGC) (Hewlett Packard HP 6890 Series, USA) with a DB-5 mass spectrometer (MS) column (60 m, 0.25 mm i.d., 0.25 μm film thickness; J&W Scientific). The temperature program was: 90 °C (2 min hold); 25 °C/min increase to 180 °C (2 min hold); 1.5 °C/min increase to 220 °C (2 min hold); and 3 °C/min increase to 275 °C (12 min hold) and 25 °C/min increase to 300 °C (4 min hold). The total run time was 71.6 min.

Detection of BDE-28, -47, -99, -100, -153, -154 and -183, and HBCD were conducted on a HRGC (Agilent 6890 Series GC system, USA) coupled with a low-resolution MS (LRMS) (Agilent 5973 Network Mass Selective Detector, USA) configured with a split/split-less injector (Agilent Technologies, Santa Clara, USA) and a DB-5 MS column (30 m, 0.25 mm i.d., 0.25 μm film thickness; J&W Scientific). The temperature program was 90 °C (1 min hold); 25 °C/min increase to 180 °C; 2.5 °C/min increase to 220 °C (1 min hold); and 20 °C/min increase to 320 °C (10 min hold). The total run time was 36.6 min. For BDE-206, -207, -208 and -209, a HRGC (Agilent 6890 Series GC system, USA) coupled with a LRMS (Agilent 5973 Network Mass Selective Detector, USA) configured with a programmable temperature vaporization (PTV) injector (Agilent Technologies) was used. Separation was performed on a DB-5-MS column (10 m, 0.25 mm i.d., 0.10 μm film thickness; J&W Scientific, Agilent Technologies). The temperature program was 80 °C (2 min hold); 30 °C/min increase to 315 °C (6 min hold). The total run time was 15.83 min. BDE-28, -183, -206, -207, and -208 were not originally in the POPs mixture, but measured due to possible de-bromination of the original PBDEs.

The target ions for all analyzed PCBs, OCPs and BFRs are presented in Table 1.

**Table 1.** Overview of target ions (m/z, for PCBs and OCPs), precursor and product ions (m/z, for BFRs and PFASs), limit of detection (LOD; ng/g wet weight) and relative recovery (%) of PCBs, OCPs, BFRs and PFASs measured in pooled liver tissue from CD-1 mice. PCBs, OCPs and BFRs were analyzed by electron capture negative ionization (ECNI) in selected ion monitoring (SIM) mode. PFASs were analyzed in electrospray ionization (ESI) and negative ionization mode.

| Compounds | Target ion | LOD | Recovery |
| --- | --- | --- | --- |
| *Polychlorinated biphenyls (PCBs)* |  |  |  |
| PCB-52 | 291.9 | 0.973 | 87 |
| PCB-101 | 325.9 | 0.088 | 99 |
| PCB-118 | 325.9 | 0.011 | 96 |
| PCB-138 | 359.9 | 0.021 | 97 |
| PCB-153 | 359.9 | 0.007 | 90 |
| PCB-180 | 395.8 | 0.008 | 102 |
| *Organochlorine pesticides (OCPs)* |  |  |  |
| HCB | 283.8 | 0.007 | 98 |
| α-Chlordane | 409.8 | 0.008 | 99 |
| Oxychlordane | 351.8 | 0.059 | 123 |
| *Trans*-nonachlor | 443.8 | 0.007 | 121 |
| α-HCH | 71 | 0.025 | 108 |
| β-HCH | 71 | 0.052 | 121 |
| γ-HCH | 71 | 0.020 | 116 |
| *p,p'*-DDE | 317.9 | 0.164 | 133 |
| *Brominated flame retardants (BFRs)* |  |  |  |
| BDE-28 | 79/81 | 0.033 | 86 |
| BDE-47 | 79/81 | 0.098 | 86 |
| BDE-99 | 79/81 | 0.069 | 117 |
| BDE-100 | 79/81 | 0.051 | 106 |
| BDE-153 | 79/81 | 0.131 | 108 |
| BDE-154 | 79/81 | 0.132 | 92 |
| BDE-183 | 79/81 | 0.232 | 94 |
| BDE-206 | 484/486 | 0.093 | 156 |
| BDE-207 | 484/486 | 0.069 | 121 |
| BDE-208 | 484/486 | 0.051 | 129 |
| BDE-209 | 495/497 | 0.224 | 120 |
| HBCD | 79/81 | 1.182 | 155 |
| *Perfluoroalkylated substances (PFASs)* |  |  |  |
| PFHxS | 398.9/80 | 0.220 | 97 |
| PFOS | 498.9/99 | 0.130 | 93 |
| PFOA | 413/369 | 0.240 | 105 |
| PFNA | 463/419 | 0.180 | 99 |
| PFDA | 513/469 | 0.150 | 100 |
| PFUnDA | 563/519 | 0.090 | 97 |

### **Analysis of PFASs**

The analysis of PFASs, originally described by Grønnestad and colleagues [7], is briefly described below.

Approximately 0.20-0.55 g homogenized liver tissue (homogenized by a T25 Ika Ultra-Turrax^®^) was spiked with internal standards (a ^13^C-labeled perfluoroalkyl mix obtained from Wellington Laboratories, Guelph, Canada) and extracted twice with methanol. Lipids were removed using activated carbon (EnviCarb). Quantification of PFHxS, PFOS, PFOA, PFNA, PFDA and PFUnDA was carried out using a high-performance liquid chromatography (HPLC) with a Discovery C18 column, connected to a C18 pre-column (Supelco, Sigma-Aldrich, Oslo, Norway) and a liquid chromatography (LC) tandem MS (MS-MS) (API 3000, LC/MS/MS system). Target ions are presented in Table 1.

### **QA/QC OCPs, PCBs, BFRs and PFASs**

One non-spiked and two spiked samples of commercial cod oil, three blanks (solvents) and one harp seal blubber (*Pagophilus groendlandicus*) were included as internal reference material (IRM) for the analysis of OCPs, PCBs and BFRs. For analyses of PFASs, the analytical series included one non-spiked (blind) and two spiked salmon liver samples and three blanks (solvents).

The LOD was defined as three times the noise level for each compound. LOD and relative recovery are presented in Table 1. *p,p’*-DDE, BDE-206 and HBCD were corrected for high relative recovery (> 130%). Percentage lipid used for adjusting the concentrations of the lipid-soluble PCBs, OCPs and BFRs are shown in S2 Table. Positive procedural blanks were found for some compounds. Results were corrected for blanks, if the blank concentrations were consistent for all solvent samples. The analytical quality was approved by satisfactory quality control measures, and results were within the accepted ranges for the analyzed CRM (2525: Fish containing PCBs, OCPs, BFRs (Cerilliant Corporation, Round Rock, USA) and 350: Fish oil containing PCBs (Commission of the European Communities, Belgium)) and inter-laboratory tests (AMAP: human blood containing PCBs, OCPs, BFRs, PFASs (Québec, Canada)).

## **Liver microsomal preparations and cytochrome P450 activity**

Hepatic microsomes were prepared as described in Rasmussen and colleagues [8]. In brief, liver was homogenized in Tris-sucrose buffer (10 mM Tris-HCl, 250 mM sucrose, pH 7.4) at a 1:2 ratio (weight of sample:volume of buffer) and centrifuged for 10 min (at 10 000 g and 4 °C using an Eppendorf Centrifuge 5417R). The supernatant was extracted and diluted with TRIS-sucrose buffer, containing 8 mM CaCl_2_, to a final volume of 25 mL. Samples were centrifuged (30 min at 25 000 g and 4 °C, Beckman Coulter OptimaTML-80 XP Ultracentrifuge) after 4 min incubation on ice. Experimental conditions for the activity assay are reported in Table 2.

**Table 2.** Experimental conditions for cytochrome P450 (CYP) enzyme activity assays.

| CYP isoform | Reaction | Substrate | Substrate final concentration (μM) | Microsomal protein (mg) | NADPH (mM) | Incubation time (min) | Terminating reagent |
| --- | --- | --- | --- | --- | --- | --- | --- |
| CYP1A1 | EROD | 7-ethoxyresorufin | 1 | 0.2 | 0.5 | 5 | 100 % Methanol |
| CYP1A | MROD | 7-methoxyresorufin | 2 | 0.2 | 0.5 | 7 | 100 % Methanol |
| CYP3A11 | BROD | 7-benzyloxyresorufin | 2 | 0.2 | 0.5 | 7 | 100 % Methanol |
| CYP2B10 | PROD | 7-pentoxyresorufin | 10 | 0.2 | 0.5 | 20 | 100 % Methanol |
| CYP2A5 | CoH | coumarin | 200 | 0.2 | 0.5 | 15 | 100% Methanol |
| CYP2E1 | PNPH | p-nitrophenol | 200 | 0.5 | 0.5 | 30 | 40% TCA |

Abbreviations: EROD = 7-ethoxyresorufin O-deethylase; MROD = 7-methoxyresorufin O-demethylase; BROD = 7-benzyloxyresorufin O-dealkylation; PROD = 7-pentoxyresorufin O-depenthylase; CoH = coumarin hydroxylase; PNPH = p-nitrophenol hydroxylase.

HPLC analyses of CYP enzyme activities were performed on a system comprising a pump (L-7100), autosampler (L-7200), fluorescence (L-7485) or UV-Vis (L-4250) detector, and D-7000 HPLC Manager software (Merck-Hitachi, Tokyo, Japan). A Hypersil ODS (3 µm, 60 x 4.6 mm), with a guard column, was used for quantification of resorufin and coumarin. For p-nitrocatechol, a Lichrosphere RP-18 column (5 µm, 250 x 4 mm) with a guard column, was used.

# **References**

1. Berntsen HF, Berg V, Thomsen C, Ropstad E, Zimmer KE. The design of an environmentally relevant mixture of persistent organic pollutants for use in *in vivo* and *in vitro* studies. J Toxicol Env Heal A. 2017;80:1002-16. doi: 10.1080/15287394.2017.1354439.

2. Secretariat of the Stockholm Convention [Internet]. All POPs Listed in the Stockholm Convention. 2019 [cited 02 April 2020]. Available from: http://chm.pops.int/TheConvention/ThePOPs/AllPOPs/tabid/2509/Default.aspx.

3. Houde M, De Silva AO, Muir DCG, Letcher RJ. Monitoring of perfluorinated compounds in aquatic biota: an updated review. Environ Sci Technol. 2011;45(19):7962-73. doi: 10.1021/es104326w.

4. Olsen GW. PFAS biomonitoring in higher exposed populations. In: DeWitt JC, editor. Toxicological effects of perfluoroalkyl and polyfluoroalkyl substances Molecular and Integrative Toxicology. Humana Press, Cham; 2015. p. 77-125. doi: 10.1007/978-3-319-15518-0_4.

5. Brevik EM. Gas chromatographic method for the determination of organochlorine pesticides in human milk. Bull Environ Contam Toxicol. 1978;78:281-6. doi: 10.1007/BF01685799.

6. Polder A, Müller MB, Lyche JL, Mdegela RH, Nonga HE, Mabiki FP, et al. Levels and patterns of persistent organic pollutants (POPs) in tilapia (*Oreochromis sp.*) from four different lakes in Tanzania: geographical differences and implications for human health. Sci Total Environ. 2014;488-489:252-60. doi: 10.1016/j.scitotenv.2014.04.085.

7. Grønnestad R, Villanger GD, Polder A, Kovacs KM, Lydersen C, Jenssen BM, et al. Maternal transfer of perfluoroalkyl substances in hooded seals. Environ Toxicol Chem. 2017;36(3):763-70. doi: 10.1002/etc.3623.

8. Rasmussen MK, Ekstrand B, Zamaratskaia G. Comparison of cytochrome P450 concentrations and metabolic activities in porcine hepatic microsomes prepared with two different methods. Toxicol In vitro. 2011;25(1):343-6. doi: 10.1016/j.tiv.2010.10.007.
