# Supplementary material for: Perinatal exposure to a human relevant mixture of persistent organic pollutants: Effects on mammary gland development, ovarian folliculogenesis and liver in CD-1 mice
Source: PLoS One. 2021 Jun 10;16(6):e0252954. doi: 10.1371/journal.pone.0252954 (PMC8191980; doi:10.1371/journal.pone.0252954)
Supplement: S1 Table — Individual PCBs, OCPs, BFRs and PFASs in pooled liver samples of dietary exposed pregnant (gestation d 17) and post-pregnant (21 d post-partum) dams and maternally exposed female offspring (3, 6 and 9 weeks of age). Mice were exposed to the mixture of POPs at Control, Low or High doses (0x, 5000x or 100 000x human estimated daily intake, respectively). Values are presented as ng/g lipid weight for PCBs, OCPs and BFRs, and ng/g wet weight for PFASs. (DOCX) [file pone.0252954.s003.docx]

**S1 Table. Lipid adjusted concentrations of POPs.** Individual PCBs, OCPs, BFRs and PFASs in pooled liver samples of dietary exposed pregnant (gestation d 17) and post-pregnant (21 d post-partum) dams and maternally exposed female offspring (3, 6 and 9 weeks of age). Mice were exposed to the mixture of POPs at Control, Low or High doses (0x, 5000x or 100 000x human estimated daily intake, respectively). Values are presented as ng/g lipid weight for PCBs, OCPs and BFRs, and ng/g wet weight for PFASs.

|  | *Pregnant dams* | | | *Post-pregnant dams* | | | *3 weeks offspring* | | | *6 weeks offspring* | | | *9 weeks offspring* | | |
| --- | --- | --- | --- | --- | --- | --- | --- | --- | --- | --- | --- | --- | --- | --- | --- |
| Compounds | Control | Low | High | Control | Low | High | Control | Low | High | Control | Low | High | Control | Low | High |
| *Polychlorinated biphenyls (PCBs)* | | | |  |  |  |  |  |  |  |  |  |  |  |  |
| PCB-52 | <LOD | <LOD | <LOD | <LOD | <LOD | <LOD | <LOD | <LOD | <LOD | <LOD | <LOD | <LOD | <LOD | <LOD | <LOD |
| PCB-101 | <LOD | 158.2 | 1725.0 | <LOD | 124.7 | 1385.7 | <LOD | 120.1 | 1050.5 | <LOD | 5.7 | 36.5 | <LOD | <LOD | <LOD |
| PCB-118 | 1.3 | 908.9 | 13030.8 | 0.9 | 358.8 | 7760.9 | 2.0 | 663.7 | 16437.0 | 1.7 | 140.2 | 5450.7 | 1.8 | 39.2 | 2549.8 |
| PCB-138 | 58.2 | 35563.2 | 39569.4 | 11.6 | 5104.1 | 19050.3 | 31.9 | 14406.1 | 36625.9 | 44.9 | 7806.7 | 26077.2 | 58.6 | 3912.3 | 16795.2 |
| PCB-153 | 8.2 | 2115.1 | 22615.9 | 1.7 | 682.1 | 15967.5 | 5.6 | 1671.7 | 27009.3 | 5.7 | 656.9 | 13241.5 | 6.4 | 493.4 | 7559.4 |
| PCB-180 | 18.3 | 6945.2 | 10070.5 | 6.1 | 1517.2 | 8577.7 | 8.2 | 2767.3 | 8845.1 | 12.0 | 1534.2 | 5557.9 | 13.1 | 872.4 | 3373.0 |
| Σ6PCBs | 87.1 | 45691.6 | 87012.6 | 21.4 | 7787.9 | 52743.1 | 48.8 | 19629.9 | 89968.8 | 65.3 | 10144.7 | 50364.8 | 81.0 | 5318.4 | 30278.5 |
| *Organochlorine pesticides (OCPs)* | | | |  |  |  |  |  |  |  |  |  |  |  |  |
| HCB | 8.5 | 901.4 | 14477.2 | 3.6 | 281.4 | 6439.8 | 8.7 | 757.5 | 16181.9 | 25.4 | 201.2 | 4452.5 | 28.8 | 132.6 | 2131.6 |
| α-Chlordane | <LOD | 27.7 | 133.5 | <LOD | 20.0 | 71.6 | 0.2 | 2.2 | 14.1 | <LOD | <LOD | <LOD | <LOD | <LOD | <LOD |
| Oxychlordane | <LOD | 397.7 | 5894.3 | <LOD | 96.9 | 2216.9 | <LOD | 314.3 | 7627.2 | 1.8 | 108.9 | 3084.8 | 2.1 | 102.7 | 1563.2 |
| *Trans*-Nonachlor | 0.7 | 283.6 | 5414.6 | 0.4 | 115.5 | 3045.6 | 0.9 | 238.4 | 7225.5 | 0.8 | 72.4 | 2185.7 | 0.7 | 62.8 | 924.9 |
| α-HCH | <LOD | 76.4 | 1014.6 | <LOD | 62.4 | 873.6 | 0.9 | 61.4 | 854.0 | 0.8 | 1.9 | 11.7 | 0.8 | 0.9 | 3.1 |
| β-HCH | <LOD | 491.8 | 5593.5 | <LOD | 218.7 | 2652.1 | 1.5 | 404.3 | 6141.1 | 6.2 | 118.1 | 1657.8 | 5.7 | 58.0 | 446.0 |
| γ-HCH | <LOD | 7.3 | 58.0 | <LOD | 7.7 | 43.8 | <LOD | 1.1 | <LOD | <LOD | <LOD | <LOD | <LOD | <LOD | <LOD |
| Σ7OCPs | 9.4 | 2186.4 | 32585.7 | 4.2 | 802.6 | 15343.4 | 12.3 | 1779.2 | 38043.8 | 35.0 | 502.5 | 11392.5 | 38.1 | 357.0 | 5068.9 |
| *p,p'*-DDE | <LOD | 907.0 | 13883.9 | <LOD | 922.2 | 14189.0 | <LOD | 1200.9 | 24412.2 | <LOD | 153.6 | 1287.5 | <LOD | 10.2 | 283.7 |
| *Brominated flame retardants (BFRs)* | | | |  |  |  |  |  |  |  |  |  |  |  |  |
| BDE-28 | <LOD | <LOD | 29.6 | <LOD | 1.1 | 32.7 | <LOD | 1.1 | 54.9 | <LOD | <LOD | <LOD | <LOD | <LOD | <LOD |
| BDE-47 | <LOD | 156.7 | 3199.5 | <LOD | 189.8 | 4280.9 | <LOD | 284.7 | 7492.3 | <LOD | 24.5 | 148.2 | <LOD | <LOD | 30.1 |
| BDE-99 | <LOD | 84.5 | 1465.2 | <LOD | 83.3 | 1464.7 | <LOD | 98.1 | 1475.2 | <LOD | 19.6 | 259.0 | <LOD | 8.3 | 78.8 |
| BDE-100 | <LOD | 79.5 | 1189.1 | <LOD | <LOD | 1046.4 | <LOD | 79.3 | 1514.4 | <LOD | 20.0 | 348.8 | <LOD | 14.6 | 122.2 |
| BDE-153 | <LOD | 23.6 | 610.9 | <LOD | 35.3 | 841.3 | <LOD | 29.8 | 585.1 | <LOD | 10.0 | 270.6 | <LOD | 10.8 | 145.1 |
| BDE-154 | <LOD | 28.6 | 428.9 | <LOD | 35.7 | 509.4 | <LOD | 22.3 | 275.6 | <LOD | 4.8 | 75.8 | <LOD | 4.4 | 21.0 |
| BDE-183 | <LOD | <LOD | <LOD | <LOD | <LOD | 21.9 | <LOD | <LOD | <LOD | <LOD | <LOD | <LOD | <LOD | <LOD | <LOD |
| ΣBDE-28-183 | 0.7 | 373.2 | 6923.4 | 0.7 | 345.5 | 8197.3 | 0.7 | 515.5 | 11397.7 | 0.7 | 79.2 | 1102.7 | 0.7 | 38.5 | 397.5 |
| BDE-206 | <LOD | 12.5 | 189.1 | <LOD | 12.6 | 86.2 | <LOD | 5.7 | 95.6 | <LOD | <LOD | <LOD | <LOD | <LOD | <LOD |
| BDE-207 | <LOD | 208.6 | 2593.0 | <LOD | 103.4 | 1379.8 | <LOD | 99.0 | 2129.8 | <LOD | 8.7 | 131.3 | <LOD | 5.3 | 75.6 |
| BDE-208 | <LOD | 29.1 | 220.7 | <LOD | 8.7 | 86.2 | <LOD | 5.6 | 154.8 | <LOD | <LOD | 6.9 | <LOD | <LOD | 10.0 |
| BDE-209 | <LOD | 2010.5 | 32300.7 | 7.7 | 4409.4 | 33858.7 | 10.0 | 772.9 | 13893.9 | <LOD | 21.8 | 155.6 | 3.9 | 17.3 | 79.9 |
| ΣBDE-206-209 | 0.4 | 2260.7 | 35303.5 | 7.9 | 4534.1 | 35411.8 | 10.2 | 883.1 | 16274.1 | 0.4 | 30.6 | 293.9 | 4.1 | 22.7 | 165.6 |
| HBCD | <LOD | <LOD | <LOD | <LOD | <LOD | 40.2 | <LOD | <LOD | <LOD | <LOD | <LOD | <LOD | <LOD | 27.6 | <LOD |
| *Perfluoroalkylated substances (PFASs)* | | | |  |  |  |  |  |  |  |  |  |  |  |  |
| PFHxS | 0.3 | 13.1 | 359.7 | 0.4 | 22.9 | 485.3 | 0.9 | 7.1 | 160.6 | <LOD | 2.9 | 54.0 | <LOD | 1.9 | 35.4 |
| PFOS | 0.5 | 106.9 | 2968.3 | 2.3 | 332.0 | 6483.3 | 7.9 | 63.3 | 1477.8 | 1.2 | 37.7 | 718.1 | 1.8 | 28.9 | 540.9 |
| PFOA | 0.7 | 151.9 | 3751.1 | 1.3 | 259.6 | 4400.8 | 6.0 | 109.0 | 2532.0 | 1.4 | 30.3 | 697.8 | 1.3 | 22.3 | 375.5 |
| PFNA | 0.4 | 118.0 | 3294.1 | 0.9 | 198.6 | 4007.9 | 5.6 | 80.1 | 1738.9 | 0.9 | 37.5 | 760.6 | 1.1 | 31.8 | 614.8 |
| PFDA | 0.4 | 160.1 | 3570.1 | 1.0 | 275.7 | 4675.8 | 7.3 | 100.5 | 1817.7 | 1.2 | 43.2 | 860.0 | 1.3 | 40.9 | 647.9 |
| PFUnDA | 0.5 | 95.3 | 2239.8 | 0.6 | 159.8 | 2691.6 | 4.5 | 43.6 | 807.9 | 0.6 | 19.3 | 399.6 | 0.6 | 16.6 | 307.4 |
| ΣPFASs | 2.8 | 645.3 | 16183.3 | 6.5 | 1248.5 | 22744.6 | 32.2 | 403.7 | 8535.0 | 5.5 | 170.9 | 3490.1 | 6.3 | 142.3 | 2521.8 |
