# Supplementary material for: Perinatal exposure to a human relevant mixture of persistent organic pollutants: Effects on mammary gland development, ovarian folliculogenesis and liver in CD-1 mice
Source: PLoS One. 2021 Jun 10;16(6):e0252954. doi: 10.1371/journal.pone.0252954 (PMC8191980; doi:10.1371/journal.pone.0252954)
Supplement: S2 Table — PCBs, OCPs, BFRs and PFASs (ng/g wet weight) and lipid (%) in pooled liver samples of dietary exposed pregnant (gestation d 17) and post-pregnant (21 d post-partum) dams and maternally exposed female offspring (3, 6 and 9 weeks of age). Mice were exposed to the mixture of POPs at Control, Low or High doses (0x, 5000x or 100 000x human estimated daily intake, respectively). (DOCX) [file pone.0252954.s004.docx]

**S2 Table. Lipid percentage and wet weight concentrations of POPs.** PCBs, OCPs, BFRs and PFASs (ng/g wet weight) and lipid (%) in pooled liver samples of dietary exposed pregnant (gestation d 17) and post-pregnant (21 d post-partum) dams and maternally exposed female offspring (3, 6 and 9 weeks of age). Mice were exposed to the mixture of POPs at Control, Low or High doses (0x, 5000x or 100 000x human estimated daily intake, respectively).

|  | *Pregnant dams* | | | *Post-pregnant dams* | | | *3 weeks offspring* | | | *6 weeks offspring* | | | *9 weeks offspring* | | |
| --- | --- | --- | --- | --- | --- | --- | --- | --- | --- | --- | --- | --- | --- | --- | --- |
| Compounds | Control | Low | High | Control | Low | High | Control | Low | High | Control | Low | High | Control | Low | High |
| Lipid % | 4.50 | 3.73 | 6.13 | 6.17 | 6.72 | 8.93 | 4.06 | 4.34 | 5.54 | 6.15 | 5.43 | 5.52 | 6.99 | 7.75 | 7.01 |
| *Polychlorinated biphenyls (PCBs)* | | | |  |  |  |  |  |  |  |  |  |  |  |  |
| PCB-52 | <LOD | <LOD | <LOD | <LOD | <LOD | <LOD | <LOD | <LOD | <LOD | <LOD | <LOD | <LOD | <LOD | <LOD | <LOD |
| PCB-101 | <LOD | 5.90 | 105.80 | <LOD | 8.37 | 123.72 | <LOD | 5.21 | 58.16 | <LOD | 0.31 | 2.01 | <LOD | <LOD | <LOD |
| PCB-118 | 0.06 | 33.89 | 799.22 | 0.06 | 24.10 | 692.94 | 0.08 | 28.82 | 910.04 | 0.10 | 7.62 | 301.10 | 0.13 | 3.04 | 178.77 |
| PCB-138 | 2.62 | 1326.09 | 2426.92 | 0.71 | 342.85 | 1700.92 | 1.30 | 625.46 | 2027.80 | 2.76 | 424.15 | 1440.52 | 4.09 | 303.05 | 1177.52 |
| PCB-153 | 0.37 | 78.87 | 1387.11 | 0.10 | 45.82 | 1425.67 | 0.23 | 72.58 | 1495.38 | 0.35 | 35.69 | 731.47 | 0.45 | 38.22 | 529.99 |
| PCB-180 | 0.83 | 258.98 | 617.66 | 0.37 | 101.91 | 765.87 | 0.33 | 120.15 | 489.71 | 0.74 | 83.35 | 307.02 | 0.92 | 67.58 | 236.49 |
| Σ6PCBs | 4.94 | 1704.70 | 5337.68 | 2.31 | 524.02 | 4710.09 | 3.00 | 853.18 | 4983.12 | 5.01 | 512.09 | 2783.10 | 6.64 | 412.95 | 2123.83 |
| *Organochlorine pesticides (OCPs)* | | | |  |  |  |  |  |  |  |  |  |  |  |  |
| HCB | 0.38 | 33.61 | 887.93 | 0.22 | 18.90 | 574.98 | 0.35 | 32.89 | 895.92 | 1.56 | 10.93 | 245.96 | 2.01 | 10.27 | 149.45 |
| α-Chlordane | <LOD | 1.03 | 8.19 | <LOD | 1.34 | 6.40 | 0.01 | 0.10 | 0.78 | <LOD | <LOD | <LOD | <LOD | <LOD | <LOD |
| Oxychlordane | <LOD | 14.83 | 361.52 | <LOD | 6.51 | 197.93 | <LOD | 13.65 | 422.28 | 0.11 | 5.92 | 170.41 | 0.15 | 7.96 | 109.60 |
| *Trans*-Nonachlor | 0.03 | 10.58 | 332.09 | 0.03 | 7.76 | 271.93 | 0.04 | 10.35 | 400.04 | 0.05 | 3.93 | 120.74 | 0.06 | 4.87 | 64.85 |
| α-HCH | <LOD | 2.85 | 62.23 | <LOD | 4.19 | 78.00 | 0.04 | 2.67 | 47.12 | 0.05 | 0.11 | 0.64 | 0.06 | 0.07 | 0.21 |
| β-HCH | <LOD | 18.34 | 343.07 | <LOD | 14.69 | 236.80 | 0.06 | 17.55 | 340.01 | 0.38 | 6.42 | 91.58 | 0.40 | 4.50 | 31.27 |
| γ-HCH | <LOD | 0.27 | 3.56 | <LOD | 0.52 | 3.91 | <LOD | 0.05 | <LOD | <LOD | <LOD | <LOD | <LOD | <LOD | <LOD |
| Σ7OCPs | 0.57 | 81.51 | 1998.59 | 0.41 | 53.91 | 1369.95 | 0.58 | 77.26 | 2106.17 | 2.18 | 27.34 | 629.36 | 2.71 | 27.70 | 355.41 |
| *p,p'*-DDE | <LOD | 33.82 | 851.55 | <LOD | 82.39 | 1684.95 | <LOD | 69.35 | 1797.61 | <LOD | 11.10 | 71.13 | <LOD | 0.79 | 19.86 |
| *Brominated flame retardants (BFRs)* | | | |  |  |  |  |  |  |  |  |  |  |  |  |
| BDE-28 | <LOD | <LOD | 1.81 | <LOD | 0.08 | 2.92 | <LOD | 0.05 | 3.04 | <LOD | <LOD | <LOD | <LOD | <LOD | <LOD |
| BDE-47 | <LOD | 5.84 | 196.24 | <LOD | 12.75 | 382.23 | <LOD | 12.36 | 414.81 | <LOD | 1.33 | 8.19 | <LOD | <LOD | 2.11 |
| BDE-99 | <LOD | 3.15 | 89.87 | <LOD | 5.59 | 130.78 | <LOD | 4.26 | 81.67 | <LOD | 1.06 | 14.31 | <LOD | 0.65 | 5.52 |
| BDE-100 | <LOD | 2.97 | 72.93 | <LOD | <LOD | 93.43 | <LOD | 3.44 | 83.85 | <LOD | 1.09 | 19.27 | <LOD | 1.13 | 8.57 |
| BDE-153 | <LOD | 0.88 | 37.47 | <LOD | 2.37 | 75.12 | <LOD | 1.30 | 32.39 | <LOD | 0.55 | 14.95 | <LOD | 0.84 | 10.17 |
| BDE-154 | <LOD | 1.07 | 26.31 | <LOD | 2.40 | 45.48 | <LOD | 0.97 | 15.26 | <LOD | 0.26 | 4.19 | <LOD | 0.34 | 1.47 |
| BDE-183 | <LOD | <LOD | <LOD | <LOD | <LOD | 1.95 | <LOD | <LOD | <LOD | <LOD | <LOD | <LOD | <LOD | <LOD | <LOD |
| ΣBDE-28-183 | 0.75 | 14.18 | 424.85 | 0.75 | 23.47 | 731.90 | 0.75 | 22.60 | 631.26 | 0.75 | 4.56 | 61.17 | 0.75 | 3.31 | 28.11 |
| BDE-206 | <LOD | 0.47 | 11.60 | <LOD | 0.84 | 7.70 | <LOD | 0.25 | 5.30 | <LOD | <LOD | <LOD | <LOD | <LOD | <LOD |
| BDE-207 | <LOD | 7.78 | 159.04 | <LOD | 6.95 | 123.20 | <LOD | 4.30 | 117.92 | <LOD | 0.47 | 7.25 | <LOD | 0.41 | 5.30 |
| BDE-208 | <LOD | 1.09 | 13.53 | <LOD | 0.58 | 7.77 | <LOD | 0.24 | 8.57 | <LOD | <LOD | 0.38 | <LOD | <LOD | 0.71 |
| BDE-209 | <LOD | 74.97 | 1981.11 | 0.48 | 296.19 | 3023.10 | 0.41 | 33.56 | 769.24 | <LOD | 1.19 | 8.60 | 0.28 | 1.34 | 5.60 |
| ΣBDE-206-209 | 0.44 | 84.30 | 2165.28 | 0.69 | 304.56 | 3161.77 | 0.62 | 38.34 | 901.02 | 0.44 | 1.80 | 16.32 | 0.49 | 1.89 | 11.70 |
| HBCD | <LOD | <LOD | <LOD | <LOD | <LOD | 3.59 | <LOD | <LOD | <LOD | <LOD | <LOD | <LOD | <LOD | 2.14 | <LOD |
| *Perfluoroalkylated substances (PFASs)* | | | |  |  |  |  |  |  |  |  |  |  |  |  |
| PFHxS | 0.27 | 13.09 | 359.73 | 0.40 | 22.94 | 485.27 | 0.94 | 7.15 | 160.59 | <LOD | 2.86 | 53.96 | <LOD | 1.90 | 35.41 |
| PFOS | 0.54 | 106.87 | 2968.33 | 2.31 | 331.99 | 6483.30 | 7.90 | 63.35 | 1477.83 | 1.24 | 37.69 | 718.05 | 1.77 | 28.88 | 540.86 |
| PFOA | 0.70 | 151.93 | 3751.13 | 1.27 | 259.56 | 4400.77 | 6.00 | 109.05 | 2532.02 | 1.37 | 30.30 | 697.77 | 1.26 | 22.29 | 375.49 |
| PFNA | 0.44 | 118.03 | 3294.12 | 0.91 | 198.59 | 4007.86 | 5.55 | 80.09 | 1738.92 | 0.90 | 37.50 | 760.65 | 1.12 | 31.78 | 614.79 |
| PFDA | 0.38 | 160.09 | 3570.14 | 0.98 | 275.65 | 4675.84 | 7.25 | 100.45 | 1817.73 | 1.16 | 43.18 | 860.04 | 1.26 | 40.89 | 647.86 |
| PFUnDA | 0.49 | 95.28 | 2239.82 | 0.62 | 159.76 | 2691.55 | 4.54 | 43.58 | 807.88 | 0.58 | 19.32 | 399.59 | 0.61 | 16.55 | 307.39 |
| ΣPFASs | 2.80 | 645.28 | 16183.26 | 6.48 | 1248.49 | 22744.60 | 32.18 | 403.67 | 8534.98 | 5.45 | 170.85 | 3490.06 | 6.24 | 142.29 | 2521.79 |
