# Supplementary material for: Perinatal exposure to a human relevant mixture of persistent organic pollutants: Effects on mammary gland development, ovarian folliculogenesis and liver in CD-1 mice
Source: PLoS One. 2021 Jun 10;16(6):e0252954. doi: 10.1371/journal.pone.0252954 (PMC8191980; doi:10.1371/journal.pone.0252954)
Supplement: S3 Table — Body and liver weight of pregnant and post-pregnant dams, and female (3, 6 and 9 weeks of age) and male (9 and 30 weeks of age) offspring. Combined ovaries and uterus weight of female offspring (6 and 9 weeks of age) is also presented. Dams were dietary exposed to a mixture of POPs at Control, Low or High doses (0x, 5000x or 100 000x human estimated daily intake, respectively). Offspring were exposed in utero and through lactation (ending at 3 weeks). Pregnant dams were euthanized on gestation d 17 (n = 12, 16 and 8 for Control, Low and High, respectively). Post-pregnant dams were euthanized 21 d post-partum (n = 14, 10 and n = 11 for Control, Low and High, respectively). Females 3 weeks: n = 12 (all groups). Females 6 and 9 weeks: n = 14 (all groups, except for ovary + uterus weight from 6 weeks Control where n = 13). Males 9 and 30 weeks: n = 15 (all groups, except for liver weight from 9 weeks Low where n = 14). Results are presented as least square mean ± standard error. Bold marks significant differences (p ≤ 0.05) from Control. (DOCX) [file pone.0252954.s005.docx]

**S3 Table. Biometrical measurements**. Body and liver weight of pregnant and post-pregnant dams, and female (3, 6 and 9 weeks of age) and male (9 and 30 weeks of age) offspring. Combined ovaries and uterus weight of female offspring (6 and 9 weeks of age) is also presented. Dams were dietary exposed to a mixture of POPs at Control, Low or High doses (0x, 5000x or 100 000x human estimated daily intake, respectively). Offspring were exposed in utero and through lactation (ending at 3 weeks). Pregnant dams were euthanized on gestation d 17 (n = 12, 16 and 8 for Control, Low and High, respectively). Post-pregnant dams were euthanized 21 d post-partum (n = 14, 10 and n = 11 for Control, Low and High, respectively). Females 3 weeks: n = 12 (all groups). Females 6 and 9 weeks: n = 14 (all groups, except for ovary + uterus weight from 6 weeks Control where n = 13). Males 9 and 30 weeks: n = 15 (all groups, except for liver weight from 9 weeks Low where n = 14). Results are presented as least square mean ± standard error. Bold marks significant differences (p ≤ 0.05) from Control.

|  | Body weight (g) | Liver weight (g) | Ovaries and uterus weight (g) |
| --- | --- | --- | --- |
| *Pregnant dams* |  |  |  |
| Control | 47.40 ± 1.41 | 2.27 ± 0.06 | − |
| Low | 50.14 ± 1.22 | 2.36 ± 0.06 | − |
| High | 46.87 ± 1.82 | **2.98 ± 0.08** | − |
| *Post-pregnant dams* |  |  |  |
| Control | 38.43 ± 0.98 | 2.61 ± 0.07 | − |
| Low | 38.73 ± 1.13 | 2.75 ± 0.09 | − |
| High | 36.89 ± 0.97 | **3.34 ± 0.08** | − |
| *3 weeks offspring females* |  |  |  |
| Control | 11.82 ± 0.27 | 0.78 ± 0.04 | − |
| Low | 11.87 ± 0.28 | 0.83 ± 0.04 | − |
| High | 11.43 ± 0.27 | 0.87 ± 0.04 | − |
| *6 weeks offspring females* |  |  |  |
| Control | 24.78 ± 0.34 | 1.26 ± 0.02 | 0.22 ± 0.01 |
| Low | 24.34 ± 0.34 | 1.25 ± 0.02 | 0.24 ± 0.01 |
| High | 24.17 ± 0.35 | **1.35 ± 0.02** | 0.20 ± 0.01 |
| *9 weeks offspring females* | |  |  |
| Control | 27.86 ± 0.80 | 1.34 ± 0.03 | 0.22 ± 0.02 |
| Low | 27.70 ± 0.88 | 1.37 ± 0.03 | 0.21 ± 0.02 |
| High | 27.39 ± 0.82 | 1.43 ± 0.03 | 0.21 ± 0.02 |
| *9 weeks offspring males* |  |  |  |
| Control | 37.30 ± 1.06 | 1.98 ± 0.04 | − |
| Low | **41.17 ± 1.00** | 1.97 ± 0.04 | − |
| High | 40.58 ± 1.02 | 2.03 ± 0.04 | − |
| *30 weeks offspring males* |  |  |  |
| Control | 48.29 ± 1.29 | 2.14 ± 0.05 | − |
| Low | 48.67 ± 1.69 | 2.24 ± 0.05 | − |
| High | − | − | − |
