# Supplementary material for: Perinatal exposure to a human relevant mixture of persistent organic pollutants: Effects on mammary gland development, ovarian folliculogenesis and liver in CD-1 mice
Source: PLoS One. 2021 Jun 10;16(6):e0252954. doi: 10.1371/journal.pone.0252954 (PMC8191980; doi:10.1371/journal.pone.0252954)
Supplement: S4 Table — The number of live offspring produced by dams during dietary exposure to a mixture of POPs at Control, Low or High doses (0x, 5000x or 100 000x human estimated daily intake, respectively) were counted on gestation d 17. Live offspring of the post-pregnant dams were counted, and gender was determined, at 21 d post-partum. Results are presented as mean ± standard error. The numbers of males/females are given as the total number within each dose. (DOCX) [file pone.0252954.s006.docx]

**S4 Table. Number of live offspring, males and females.** The number of live offspring produced by dams during dietary exposure to a mixture of POPs at Control, Low or High doses (0x, 5000x or 100 000x human estimated daily intake, respectively) were counted on gestation d 17. Live offspring of the post-pregnant dams were counted, and gender was determined, at 21 d post-partum. Results are presented as mean ± standard error. The numbers of males/females are given as the total number within each dose.

|  | Live offspring | Males/Females |
| --- | --- | --- |
| *Pregnant dams* |  |  |
| Control | 11.42 ± 0.58 (n = 12) | − |
| Low | 12.81 ± 0.67 (n = 16) | − |
| High | 9.13 ± 1.64 (n = 8) | − |
| *Post-pregnant dams* |  |  |
| Control | 12.50 ± 0.65 (n = 14) | 83/81 |
| Low | 12.10 ± 0.62 (n = 10) | 63/47 |
| High | 11.09 ± 0.62 (n = 11) | 47/65 |
