# Supplementary material for: Perinatal exposure to a human relevant mixture of persistent organic pollutants: Effects on mammary gland development, ovarian folliculogenesis and liver in CD-1 mice
Source: PLoS One. 2021 Jun 10;16(6):e0252954. doi: 10.1371/journal.pone.0252954 (PMC8191980; doi:10.1371/journal.pone.0252954)
Supplement: S5 Table — Mammary gland morphology in 3, 6 and 9-week-old female CD-1 mice maternally exposed to a mixture of POPs at Control, Low or High doses (0x, 5000x or 100 000x human estimated daily intake, respectively). Qualitative development scores were conducted on whole mounts of the 4th mammary gland from the right side using a scale from 1 to 4 (1 = poor development, 4 = best development). Terminal end buds (TEBs) were defined as ends with a diameter of ≥ 100 μm. Branching density, sum of the number of intersections, mammary epithelial area and length were calculated using Sholl analysis in ImageJ software. Glandular area was calculated using ImageJ on one section of fixated 4th mammary gland from the left side. Results are presented as mean ± standard error. At 3 weeks of age, n = 11 for the High group and n = 12 for the Control and Low groups except glandular area were n = 11. At 6 weeks, n = 12 for the Control group and n = 14 for the Low and High groups except glandular area were n = 12. At 9 weeks, n = 13 for the Control and High groups, except Control group glandular area were n = 12. Furthermore, n = 14 for the Low group except glandular area were n = 12. Numbers in bold mark significant difference from Control (p ≤ 0.05). P-values ≤ 0.10 are marked with *. (DOCX) [file pone.0252954.s007.docx]

**S5 Table. Complete results on mammary gland histology.** Mammary gland morphology in 3, 6 and 9-week-old female CD-1 mice maternally exposed to a mixture of POPs at Control, Low or High doses (0x, 5000x or 100 000x human estimated daily intake, respectively). Qualitative development scores were conducted on whole mounts of the 4^th^ mammary gland from the right side using a scale from 1 to 4 (1 = poor development, 4 = best development). Terminal end buds (TEBs) were defined as ends with a diameter of ≥ 100 µm. Branching density, sum of the number of intersections, mammary epithelial area and length were calculated using Sholl analysis in ImageJ software. Glandular area was calculated using ImageJ on one section of fixated 4^th^ mammary gland from the left side. Results are presented as mean ± standard error. At 3 weeks of age, n = 11 for the High group and n = 12 for the Control and Low groups except glandular area were n = 11. At 6 weeks, n = 12 for the Control group and n = 14 for the Low and High groups except glandular area were n = 12. At 9 weeks, n = 13 for the Control and High groups, except Control group glandular area were n = 12. Furthermore, n = 14 for the Low group except glandular area were n = 12. Numbers in bold mark significant difference from Control (p ≤ 0.05). P-values ≤ 0.10 are marked with *.

|  | Development score | Terminal end buds | Branching density (N/mm^2^) | Sum of intersections (N) | Mammary epithelial area (mm^2^) | Mammary epithelial length (mm) | Glandular area (%) |
| --- | --- | --- | --- | --- | --- | --- | --- |
| *3 weeks* | |  |  |  |  |  |  |
| Control | 2.63 ± 0.25 | 10.67 ± 2.40 | 19.95 ± 0.72 | 217.92 ± 0.09 | 11.40 ± 2.19 | 3.77 ±0.35 | 0.45 ± 0.19 |
| Low | 2.38 ± 0.25 | 5.08 ± 1.38 | 19.16 ± 0.67 | 185.50 ± 18.21 | 9.91 ± 1.10 | 3.96 ± 0.25 | 0.31 ± 0.11 |
| High | 1.95 ± 0.23* | **3.17 ± 1.03** | 18.64 ± 0.74 | 146.55 ± 12.46 | 8.18 ± 0.91 | 3.45 ± 0.1 | 0.28 ± 0.11 |
| *6 weeks* | |  |  |  |  |  |  |
| Control | 2.42 ± 0.16 | 13.50 ± 1.06 | 17.63 ± 0.37 | 3240.92 ± 134.00 | 188.35 ± 4.83 | 19.32 ± 0.51 | 2.03 ± 0.22 |
| Low | 2.39 ± 0.25 | 11.36 ± 1.04 | 17.70 ± 0.68 | 2934.29 ± 189.06 | **168.79 ± 6.86** | 19.90 ± 0.80 | 2.61 ± 0.31* |
| High | 2.39 ± 0.25 | 15.86 ± 1.40 | **20.68 ± 0.54** | 3626.86 ± 198.31 | 179.73 ± 8.44 | 20.75 ± 0.48 | 2.33 ± 0.26 |
| *9 weeks* | |  |  |  |  |  |  |
| Control | 2.54 ± 0.28 | 1.62 ± 0.91 | 23.00 ± 0.67 | 5757.08 ± 472.73 | 253.88 ± 17.63 | 29.36 ± 0.97 | 2.54 ± 0.45 |
| Low | 2.39 ± 0.22 | 0.00 ± 0.00* | 24 58 ± 0.83 | 4750.14 ± 150.76 | **199.20 ± 6.19** | **26.48 ± 0.56** | 4.40 ± 0.92 |
| High | 2.85 ± 0.21 | 1.69 ± 0.99 | 25.26 ± 0.86* | 5669.31 ± 301.35 | 229.81 ± 0.23 | 27.65 ± 0.51 | 3.20 ± 0.37 |
