# Supplementary material for: Perinatal exposure to a human relevant mixture of persistent organic pollutants: Effects on mammary gland development, ovarian folliculogenesis and liver in CD-1 mice
Source: PLoS One. 2021 Jun 10;16(6):e0252954. doi: 10.1371/journal.pone.0252954 (PMC8191980; doi:10.1371/journal.pone.0252954)
Supplement: S6 Table — Severity of extramedullary hematopoiesis and centrilobular hypertrophy of hepatocytes in livers from dams (pregnant sampled at gestation d 17, and post-pregnant sampled at 21 d post-partum), and female (sampled at 3, 6 and 9 weeks of age) and male (sampled at 9 and 30 weeks of age) offspring. Dams were dietary exposed, and offspring were maternally exposed, to a mixture of POPs at Control, Low or High doses (0x, 5000x or 100 000x human estimated daily intake, respectively). Results are presented as mean ± standard error. Severity was graded on a scale from 0 to 4 (0 = no, 1 = minimal, 2 = mild, 3 = moderate, and 4 = severe change). n = 12, 14 and 14 for female offspring in all groups at 3, 6 and 9 weeks, respectively. n = 15 for male offspring in all groups at both sampling times. n = 12, 16 and 8, and n = 14, 10 and 11 for the Control, Low and High groups of pregnant and post-pregnant dams, respectively. Bold indicates significant (p ≤ 0.05) difference from Control. (DOCX) [file pone.0252954.s008.docx]

**S6 Table. Hepatic extramedullary hematopoiesis and centrilobular hypertrophy.** Severity of extramedullary hematopoiesis and centrilobular hypertrophy of hepatocytes in livers from dams (pregnant sampled at gestation d 17, and post-pregnant sampled at 21 d post-partum), and female (sampled at 3, 6 and 9 weeks of age) and male (sampled at 9 and 30 weeks of age) offspring. Dams were dietary exposed, and offspring were maternally exposed, to a mixture of POPs at Control, Low or High doses (0x, 5000x or 100 000x human estimated daily intake, respectively). Results are presented as mean ± standard error. Severity was graded on a scale from 0 to 4 (0 = no, 1 = minimal, 2 = mild, 3 = moderate, and 4 = severe change). n = 12, 14 and 14 for female offspring in all groups at 3, 6 and 9 weeks, respectively. n = 15 for male offspring in all groups at both sampling times. n = 12, 16 and 8, and n = 14, 10 and 11 for the Control, Low and High groups of pregnant and post-pregnant dams, respectively. Bold indicates significant (p ≤ 0.05) difference from Control.

|  | Extramedullary hematopoiesis | Centrilobular hypertrophy |
| --- | --- | --- |
| *Pregnant dams* |  |  |
| Control | 0.00 ± 0.00 | 0.25 ± 0.13 |
| Low | 0.00 ± 0.00 | **1.00 ± 0.13** |
| High | 0.13 ± 0.13 | **1.75 ± 0.16** |
| *Post-pregnant dams* |  |  |
| Control | 0.00 ± 0.00 | 0.29 ± 0.13 |
| Low | 0.00 ± 0.00 | **1.30 ± 0.21** |
| High | 0.00 ± 0.00 | **3.09 ± 0.21** |
| *3 weeks offspring females* |  |  |
| Control | 1.71 ± 0.21 | 0.00 ± 0.00 |
| Low | 1.29 ± 0.23 | 0.07 ± 0.08 |
| High | 1.79 ± 0.23 | **1.71 ± 0.21** |
| *6 weeks offspring males* |  |  |
| Control | 0.64 ± 0.17 | 0.00 ± 0.00 |
| Low | 0.43 ± 0.17 | **2.00 ± 0.21** |
| High | 0.50 ± 0.14 | **3.00 ± 0.21** |
| *9 weeks offspring females* |  |  |
| Control | 0.00 ± 0.00 | 0.07 ± 0.07 |
| Low | 0.14 ± 0.10 | **1.79 ± 0.30** |
| High | 0.00 ± 0.00 | **3.79 ± 0.11** |
| *9 weeks offspring males* |  |  |
| Control | 0.00 ± 0.00 | 0.20 ± 0.11 |
| Low | 0.00 ± 0.00 | **1.67 ± 0.16** |
| High | 0.00 ± 0.00 | **2.67 ± 0.16** |
| *30 weeks offspring males* |  |  |
| Control | 0.00 ± 0.00 | 0.20 ± 0.14 |
| Low | 0.00 ± 0.00 | **0.93 ± 0.21** |
| High | − | − |
